# Optimizing tylosin dosage for co-infection of *Actinobacillus pleuropneumoniae* and *Pasteurella multocida* in pigs using pharmacokinetic/pharmacodynamic modeling

**DOI:** 10.3389/fphar.2023.1258403

**Published:** 2023-09-22

**Authors:** Eon-Bee Lee, Muhammad Aleem Abbas, Jonghyun Park, Dereje D. Tassew, Seung-Chun Park

**Affiliations:** ^1^ Laboratory of Veterinary Pharmacokinetics and Pharmacodynamics, College of Veterinary Medicine, Kyungpook National University, Daegu, Republic of Korea; ^2^ DIVA Bio Incorporation, Daegu, Republic of Korea; ^3^ Quest Pharmaceutical Services (QPS), Newark, DE, United States; ^4^ Cardiovascular Research Institute, Kyungpook National University, Daegu, Republic of Korea

**Keywords:** dosage optimization, *Actinobacillus pleuropneumoniae*, *Pasteurella multocida*, pharmacokinetic/pharmacodynamic modeling, Monte Carlo simulation

## Abstract

Formulating a therapeutic strategy that can effectively combat concurrent infections of *Actinobacillus pleuropneumoniae* (*A. pleuropneumoniae*) and *Pasteurella multocida* (*P. multocida*) can be challenging. This study aimed to 1) establish minimum inhibitory concentration (MIC), minimum bactericidal concentration (MBC), time kill curve, and post-antibiotic effect (PAE) of tylosin against *A. pleuropneumoniae* and *P. multocida* pig isolates and employ the MIC data for the development of epidemiological cutoff (ECOFF) values; 2) estimate the pharmacokinetics (PKs) of tylosin following its intramuscular (IM) administration (20 mg/kg) in healthy and infected pigs; and 3) establish a PK–pharmacodynamic (PD) integrated model and predict optimal dosing regimens and PK/PD cutoff values for tylosin in healthy and infected pigs. The MIC of tylosin against both 89 and 363 isolates of *A. pleuropneumoniae* and *P. multocida* strains spread widely, ranging from 1 to 256 μg/mL and from 0.5 to 128 μg/mL, respectively. According to the European Committee on Antimicrobial Susceptibility Testing (EUCAST) ECOFFinder analysis ECOFF value (≤64 µg/mL), 97.75% (87 strains) of the *A. pleuropnumoniae* isolates were wild-type, whereas with the same ECOFF value (≤64 µg/mL), 99.72% (363 strains) of the *P. multicoda* isolates were considered wild-type to tylosin. Area under the concentration time curve (AUC), T_1/2_, and C_max_ values were significantly greater in healthy pigs than those in infected pigs (13.33 h × μg/mL, 1.99 h, and 5.79 μg/mL vs. 10.46 h × μg/mL, 1.83 h, and 3.59 μg/mL, respectively) (*p* < 0.05). In healthy pigs, AUC_24 h_/MIC values for the bacteriostatic activity were 0.98 and 1.10 h; for the bactericidal activity, AUC_24 h_/MIC values were 1.97 and 1.99 h for *A. pleuropneumoniae* and *P. multocida*, respectively. In infected pigs, AUC_24 h_/MIC values for the bacteriostatic activity were 1.03 and 1.12 h; for bactericidal activity, AUC_24 h_/MIC values were 2.54 and 2.36 h for *A. pleuropneumoniae* and *P. multocida*, respectively. Monte Carlo simulation lead to a 2 μg/mL calculated PK/PD cutoff. Managing co-infections can present challenges, as it often demands the administration of multiple antibiotics to address diverse pathogens. However, using tylosin, which effectively targets both *A. pleuropneumoniae* and *P. multocida* in pigs, may enhance the control of bacterial burden. By employing an optimized dosage of 11.94–15.37 mg/kg and 25.17–27.79 mg/kg of tylosin can result in achieving bacteriostatic and bactericidal effects in 90% of co-infected pigs.

## 1 Introduction

Tylosin belongs to the macrolide family of antibiotics, which share a macrocyclic lactone ring structure critical for their antibacterial activity (Arsic et al., 2018). Variations in this ring structure can lead to differences in their spectrum of activity and pharmacokinetic properties (Lenz et al., 2021). It has been widely used to treat respiratory, skin, and gastrointestinal infections in pigs, effectively targeting bacterial pathogens like *Pasteurella multocida*, *Haemophilus parasuis*, and *Actinobacillus pleuropneumoniae* (Urbanová et al., 1975; DeRosa et al., 2000). Tylosin can be administered orally, intravenously, or intramuscularly, with its efficacy influenced by factors such as dosage, frequency of administration, and the specific bacterial strain being treated (Wu et al., 2019). Careful antibiotic use is essential to minimize antimicrobial resistance, and adherence to guidelines for responsible antibiotic use in livestock production is crucial (Lee et al., 2013). In addition to their antibacterial effects, macrolides, including tylosin, have demonstrated immunomodulatory and anti-inflammatory properties. They can hinder the production of pro-inflammatory cytokines, potentially contributing to therapeutic benefits in certain infections and non-infectious inflammatory conditions (Cao et al., 2006).

The typical recommended dosage for pigs is 20 mg of tylosin per kilogram of the pig’s body weight (Couper et al., 2006). This dosage is commonly prescribed to treat a variety of infections affecting the respiratory system, skin, and gastrointestinal tract in pigs (Huang et al., 2021; Ronaghinia et al., 2021). By administering this dosage, the aim is to effectively combat the specific bacteria responsible for the infection while also ensuring that the risk of adverse effects is kept to a minimum. The dosage is carefully selected based on factors such as the pig’s weight and the severity of the infection to ensure that it achieves optimal therapeutic outcomes without causing harm to the animal (Rizk et al., 2019).

Porcine respiratory disease complex (PRDC) is a common respiratory disease in pigs and can lead to significant economic losses in the swine industry (Eddicks et al., 2021). PRDC is caused by a combination of viral, bacterial, and environmental factors (Woeste and Grosse Beilage, 2007). Therefore, given the complexity of managing PRDC, it is common for a comprehensive and prolonged approach to be necessary. Effective antimicrobial therapy plays a crucial role in both preventing and managing multiple infections associated with PRDC (Chae, 2016).

*A. pleuropneumoniae* and *P. multocida* are two important pathogens that are commonly associated with PRDC (Cheong et al., 2017). *A. pleuropneumoniae* is a Gram-negative bacterium that is a significant cause of respiratory diseases in pigs. This bacterium can cause several clinical signs, including coughing, fever, weight loss, and pleurisy (Hälli et al., 2020). *A. pleuropneumoniae* is highly contagious and can rapidly spread through herds of pigs (Plasencia-Muñoz et al., 2020), which is associated with stressful conditions, including transportation, weaning, and changes in the herd (Loera-Muro et al., 2021). The bacterium can survive for long periods in the environment and is frequently present on farms without causing any clinical signs (Assavacheep and Rycroft, 2013). *P. multocida* is a Gram-negative bacterium that is commonly observed in the upper respiratory tract of pigs (Hamilton et al., 1998). *P. multocida* can cause several clinical signs, including fever, lethargy, anorexia, respiratory distress, and joint swelling (Giordano et al., 2015).

A mathematical model is created to explain how a drug behaves in the body [pharmacokinetics (PK)] and how it produces its effects [pharmacodynamics (PD)] (Derendorf et al., 2000). This model involves equations that relate the drug concentration in the body to the observed response, such as changes in blood pressure or inhibition of bacterial growth (Bauer and Bauer, 2008). To evaluate the likelihood of achieving the desired pharmacological response for a given drug dosing plan, PK/PD modeling uses the Monte Carlo Simulation, a powerful computational technique (Chua and Tam, 2022). The Probability of Target Attainment (PTA) is a vital concept in this analysis, as it helps assess the drug’s effectiveness and predictability (Cristinacce et al., 2019). Once the Monte Carlo Simulation is validated and reaches convergence, it yields final dose values through statistical analysis (Shen and Kuti, 2023). These values represent the average dose and uncertainty at specific points of interest, considering the intricate interactions within the system. The management of *A. pleuropneumoniae* and *P. multocida* concurrent infections can be challenging, thereby leading to difficulties in formulating a therapeutic strategy that can effectively combat all bacteria that are simultaneously present. Therefore, it is significant to employ a multifaceted PK/PD model enabling the determination of a rational dosage regimen to optimize the efficacy of a drug for a strong therapeutic effect (Derendorf et al., 2000).

The PK/PD cutoff value (CO_PD_) is a measure that can be useful in assessing the effectiveness of antibiotics in treating bacterial infections and is closely related to clinical efficacy (Rodríguez-Gascón et al., 2021). Establishing the CO_PD_ before approving an antimicrobial drug for clinical use is generally recommended (Derendorf et al., 2000). However, to our knowledge, there is currently no published data available regarding the CO_PD_ of tylosin against *A. pleuropneumoniae* and *P. multocida* co-infections in pigs.

Therefore, this study aimed to 1) establish the minimum inhibitory concentration (MIC), MBC, time kill curve, and post-antibiotic effect (PAE) of tylosin *in vitro* in the Brain Heart Infusion (BHI) against 89 *A. pleuropneumoniae* and 363 *P. multocida* pig isolates and employ the MIC data for the development of epidemiological cutoff (ECOFF) values; 2) evaluate the PK profile of tylosin following its intramuscular (IM) administration in healthy and *A. pleuropneumoniae*- and *P. multocida*-infected pigs; 3) evaluate the *ex vivo* time kill curves of tylosin plasma from healthy and infected pigs against *A. pleuropneumoniae* and *P. multocida*; 4) undertake PK–PD modeling of the 125 BHI and plasma *in vitro* time kill data used as a surrogate of *in vivo* efficacy area under the concentration time curve (AUC)_24 h_/MIC ratios to establish two levels of growth inhibition; and 5) use the PK–PD-modeled data for predicting CO_PD_ and effective dosage regimens required for achieving bacterial killing of both *A. pleuropneumoniae* and *P. multocida*.

## 2 Materials and methods

### 2.1 Chemicals and reagents

Tylosin standard and nicotinamide adenine dinucleotide (NAD) was purchased from Sigma-Aldrich (St. Louis, MO, United States). Each milliliter of the tylosin injectable solution (50 mg/mL) contains 50 mg of tylosin activity (as tylosin base) in 50% propylene glycol with 4% benzyl alcohol which acts as a preservative, preventing the growth of bacteria; water for injection was obtained from Samyang Anipharm (Seoul, Korea). BHI and Mueller Hinton Broth (MHB) were purchased from BD Company (New Jersey, United States). Sheep blood defibrinated (SBD) was acquired from Kisan Bio (Seoul, Korea).

### 2.2 Bacterial strains

A total of 89 and 363 strains of *A. pleuropneumoniae* and *P. multocida*, respectively, were provided by the Animal and Plant Quarantine Agency (Kimchen, Korea). Quality control (QC) strains American Type Culture Collection (ATCC) 27088 and ATCC 43137 for *A. pleuropneomiae* and *P. multocida*, respectively, were purchased from ATCC (Manassas, VA, United States). Each isolate strain was cultured as described in previous research with some modifications (Dorey et al., 2017). *A. pleuropneumoniae* and *P. multocida* was subcultured over three times to reach stable growth in BHI containing 0.02% NAD for *A. pleuropneumoniae* and MHB containing 2% SBD for *P. multicoda.*

### 2.3 Study animals and experimental design

Fourteen clinically healthy crossbred Duroc × (Landrace × Yorkshire) male pigs, approximately 5–6 weeks of age with an average weight of 9.5 ± 1.1 kg, were obtained from Petobio (Hanam, Gyeonggi-do, Korea) and transported to the study site at Gyeongsangbuk-do Veterinary Service Laboratory (Daegu, South Korea). The animal study was approved by the Animal Ethics Committee of the Petobio Clinical Institute (PTB-2022-IACUC-013-A). Animals were acclimatized for 1 week with free access to water and food. Following proper adaptation to diet and environment, the pigs were randomly assigned into two groups, seven for the healthy (non-infected) group (Group-1) and seven for the infected group (Group-2). All 14 clinically healthy pigs were further confirmed as negative through nasal swabs (Copan Diagnostics, Murrieta, CA, United States) using polymerase chain reaction (PCR) amplification of *apxIVA* and *kmt1* genes for detecting *A. pleuropneumoniae* and *P. multocida*, respectively. The pigs in Group-2 were manually restrained using a pig snare into a dog-sitting position with front legs extended, and each pig was intranasally inoculated with 1-mL mixed suspension (0.5 mL per naris) containing 2.0 × 10^9^ colony forming unit (CFU)/mL of *A. pleuropneumoniae* (BA2000013) (0.5 mL) and 2.0 × 10^9^ CFU/mL of *P. multocida* (BA1700127) (0.5 mL) using a syringe upon inspiration. During the experiment, clinical respiratory disease scores, appearance/abnormal signs, and clinical signs were recorded. *A. pleuropneumoniae* and *P. multocida* infections were monitored by culturing nasal swabs and confirmed by PCR amplification of *apxIVA* and *kmt1* genes for detecting *A. pleuropneumoniae* and *P. multocida,* respectively, according to a previously described method (Schaller et al., 2001; Shalaby et al., 2021). Following 12 h of infection, the pigs in both groups received 20 mg/kg tylosin by IM administration (Couper et al., 2006). Blood samples from every pig were collected from the jugular vein at 0 (pre-dose), 0.25, 0.5, 0.75, 1, 2, 4, 6, 8, 12, and 24 h following IM administration in anticoagulant-containing vacutainers (BD Company, New Jersey, United States) with anticoagulants. The blood samples were centrifuged at 3,000 rpm for 10 min, and the plasma was transferred to Eppendorf tubes and stored at −70°C until analysis.

### 2.4 *In vitro* susceptibility study and MIC, MBC, and ECOFF determination

The MIC of tylosin for 89 strains of *A. pleuropneumoniae* (NAD-containing BHI) and 363 strains of *P. multocida* (MHB containing 2% SBD) was determined using a two-fold serial dilution method with 0.25–256 μg/mL of concentration following the Clinical and Laboratory Standards Institute (CLSI) guidelines (CLSI, 2017). Those isolates with MIC values over 256 μg/mL were re-tested using a broader range of tylosin dilutions. Inoculated plates were kept at 37°C for 48 h. The MIC value was considered the lowest drug concentration that caused complete visible growth inhibition of bacteria in the medium. *A. pleuropneumoniae* (ATCC 27088) and *P. multocida* (ATCC 43137) susceptibility tests were simultaneously performed as QC strains to evaluate the results of the abovementioned susceptibility testing. MIC_50_ and MIC_90_, which inhibit bacterial growth by 50% and 90% of isolates, respectively, were determined as described in a previous study (Schwarz et al., 2010). MBC was tested by inoculating the supplemented BHI plates containing 0.02% NAD and MHB containing 2% SBD with a 20-μL suspension received from three higher concentrations greater than the initial MIC detection with no distinct bacteria and were incubated for 24 h at 37°C with 5% CO₂. These were tested to determine the MBC using the spot plate technique to determine a 3log10 reduction in the inoculum count. A strain with a MIC value similar to MIC_90_ was selected for further *in vitro* susceptibility study. MIC results were used to determine ECOFF values using the iterative statistical method processed in the ECOFFinder software (version 2.1; https://www.eucast.org/mic_distributions_and_ecoffs/, EUCAST).

### 2.5 PAE determination

Selected isolates susceptible to tylosin with the lowest possible MIC were used for PAE determination as previously described (Luo et al., 2020). Strains were cultured in BHI containing 0.02% NAD for *A. pleuropneumonia* and MHB containing 2% SBD for *P. multicoda* at 37°C to the log phase of growth to produce a final inoculum of 1.5 × 10^7^ CFU/mL. Bacteria were exposed to tylosin concentrations equal to 0.5×, 1×, or 4× MIC. Growth controls were simultaneously inoculated without antibiotics. Tubes were placed in a 37°C shaker for 2 h. At the end of the exposure period, antibiotics were washed by diluting 1:1,000 with sterile NAD-containing BHI for *A. pleuropneumoniae* and MHB broth containing 2% SBD for *P. multicoda*. Controls were handled similarly. Following dilution of the antibiotics, tube contents were incubated at 37°C until turbidity developed. Bacterial counts were determined at 0, 1, 2, 3, 4, 5, 6, and 7 h following dilution. PAE was calculated as follows: PAE = T−C, where PAE refers to the post-antibiotic effect, T is the time required for the viable counts in bacterial suspension to increase by 1 log10 above the count following tylosin removal by dilution, and C is the concentration for the viable counts in bacterial suspension to increase by 1 log10 for controls without tylosin treatment following dilution (Luo et al., 2020).

### 2.6 *In vitro* and *ex vivo* time kill curves

*In vitro* time kill curves of tylosin against *A. pleuropneumoniae* or *P. multocida* was established using the broth dilution method following the CLIS guidelines (CLSI, 2017). Briefly, the bacteria adjusted to a final inoculum of 1.5 × 10^6^ CFU/mL was exposed to various tylosin concentrations ranging from 0.5× to 4× MIC. For control growth curves, BHI and MHB without tylosin was used. Bacterial counts were performed by applying to BHI plates containing 0.02% NAD or MHB containing 2% SBD at 0, 1, 2, 4, 8, 12, and 24 h of culture following incubation for 24 h at 37°C.

*Ex vivo* time kill curves of tylosin were performed similarly to the above mentioned *in vitro* time kill curve using plasma obtained from healthy and infected pigs with *A. pleuropneumoniae* (BA2000013) and *P. multocida* (BA1700127) at 0-, 0.25-, 0.5-, 0.75-, 1-, 2-, 4-, 6-, 8-, 12-, and 24-h time points following IM injection of tylosin. The plasma at each time interval collected from the same pigs (*n* = 3) were used. Plasma samples were pre-filtered through a 0.22-μm membrane to clear any bacterial contamination. Bacteria were cultured on BHI supplemented with 0.02% NAD or MHB containing 2% SBD and incubated overnight at 37°C. 5 uL of bacterial culture in the stationary phase was introduced into 0.5 mL of plasma, resulting in a final inoculum of 1 × 10^6^ CFU/mL. Subsequently, the tubes containing the bacterial-plasma mixtures were incubated at 37°C, and bacterial counts were assessed using the plate count method at 1, 2, 4, 8, 12, and 24 h (Huang et al., 2018).

### 2.7 HPLC procedures and PK analysis of tylosin

Frozen plasma samples were thawed at room temperature, and 245 uL of plasma was transferred to new prechilled centrifuge tubes combined with 5 uL of 0.025 μg/mL as the internal standard. For drug extraction, plasma sample aliquots were mixed with methanol (2 mL). After vortexing (15 min) and centrifugation (12,000 × g, 10 min), the supernatant was separated and filtered through a 0.22-μm nylon syringe filter, and dried in a water bath using nitrogen at 50°C. The residue was dissolved in 100-uL methanol, agitated (1 min), and centrifuged (12,000 rpm, 10 min). Drug levels in the final 70 uL were determined using the liquid chromatography-tandem mass spectrometry method (Agilent 1200 HPLC system; API 4000 triple quadrupole mass spectrometer, CA, United States). The mass spectrometer was set up with an electrospray positive ionization mode (ESI+) using a capillary voltage of 3,500 V and had optimal ESI–MS parameters, including a drying gas temperature of 350°C, a drying gas flow of 5 L/min, and a nebulizing gas pressure of 45 psi. Separations were accomplished using an Eclipse plus C18 column (2.1 × 100 mm, 3.5 μm) (Agilent Technologies, CA, United States). The mobile phase consisted of a mixture of 0.1% formic acid in water (Eluent A) and 0.1% formic acid in acetonitrile solution (Eluent B) with a ratio of 30:70 (v/v) and a concentration of 1 mM. The flow rate was 0.4 mL/min, and the sample injection volume was 3 μL. The column temperature was maintained at 40°C. The monitored precursor ion for tylosin was 916.3 m/z. The validation of the assay was performed by spiked plasma samples at five different levels. The limit of detection was the concentration at which the signal-to-noise ratio was greater than three with a value of 0.017 μg/mL, whereas the limit of quantification was the concentration at which the signal-to-noise ratio was ten with a value of 0.053 μg/mL. The correlation coefficient (R) was above 0.98 in the linear range of 0.025–4 μg/mL. Inter- and intra-assay precision was determined to be all <10% and the accuracy of the assay was 101.38% ± 34.24% (Huang et al., 2018).

Tylosin time–concentration data in the plasma of individual pigs were analyzed using WinNonlin Version 8.3 software (Certara, NJ, United States) employing non-compartmental modeling. The maximal drug concentration (Cmax) was directly determined from the data with T_max_ defined as the time of the first occurrence of C_max_. To calculate the AUC, the linear trapezoidal rule was used. Additional PK parameters, including terminal half-life (T_1/2_λ_z_) and mean residence time (MRT), were also determined.

### 2.8 PK/PD integration analysis

The PK/PD integration was estimated on the basis of the area under the plasma concentration curve over 24 h divided by the MIC (AUC_24 h_/MIC) (Aliabadi and Lees, 2001). The *in vitro* drug effect E) was determined by computing the log10 difference between 0- and 24-h incubation. Data were assessed using the sigmoid E_max_ model equation as shown below:

$$E=E_{0}-\left[\frac{\left(E_{\max}\times C^{\gamma }\right)}{\left(C^{\gamma }+{EC}_{50}^{\gamma }\right)}\right]$$

where E_0_ represents drug effect, which is calculated as the change in bacterial count in the control samples after 24 h compared with the initial inoculum. E_max_ is the difference in effect between maximum growth (growth in control, E_0_) and minimum growth. EC_50_ is the AUC_24 h_/MIC value producing half reduction (50%) in bacterial counts from the initial inoculum. C represents the AUC_24 h_/MIC ratio, and γ is the Hill coefficient representing the steepness of the AUC_24 h_/MIC effect curve. T > MIC represents the time that tylosin plasma concentration is above the MIC (Turnidge, 1998).

### 2.9 Monte Carlo analysis for PK/PD cutoff values

The Monte Carlo simulation (MCS) analysis involved conducting 10,000 MCS trials on the basis of predetermined PK parameters and PK/PD targets (AUC_24 h_/MIC) that exhibited a bactericidal effect (E = −3) (Lei et al., 2018). The PK/PD cutoff value (CO_PD_) was identified as the MIC level at which the probability of target at which the probability of target attainment (PTA) reached 90%, following the previously described method (Turnidge and Paterson, 2007). The daily dose was calculated using MCS in the Oracle Ball (Oracle Corporation, Redwood Shores, CA, United States) for 10,000 iterations to estimate 50% and 90% target attainment rates for bacteriostatic and bactericidal effects.

### 2.10 Dose estimations

The calculation of the potential optimal dosage was determined using the AUC_24 h_/MIC value at different activity levels, including the bacteriostatic activity (E = 0) and bactericidal activity (E = −3) using the following equation:

$$\text{Dose}=\frac{\left(\frac{{AUC}_{24hr}}{MIC}\right)\times MIC\times Cl}{F\times fu}$$

where AUC_24 h_/MIC represents the target endpoint for optimal efficacy, MIC represents the minimum inhibitory concentration in this study, Cl refers to clearance (Prats et al., 2002), F is bioavailability, and fu indicates a free fraction of tylosin in plasma (Bauer and Bauer, 2008). Relative bioavailability can serve as a substitute when intravenous administration is not available (TOUTAIN & BOUSQUET-MÉLOU, 2004). The calculation was based on previous research (Huang et al., 2018).

### 2.11 Statistical analysis

Data were presented as means ± standard deviations. Statistical analysis was performed with Student’s t-test using GraphPad Prism software version 8.0.1 (CA, United States). *p* < 0.05 was considered statistically significant.

## 3 Results

### 3.1 MIC, MBC, and ECOFF determinations

**FIGURE 1:**
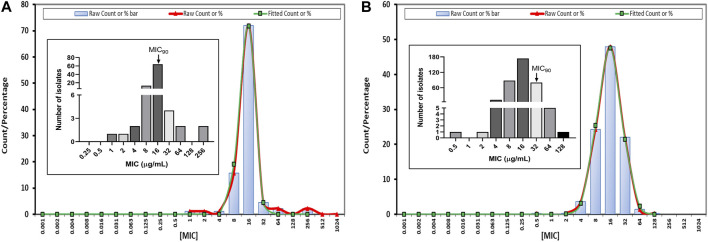
Fitted minimum inhibitory concentration (MIC) distribution for tylosin against *Actinobacillus pleuropneumoniae* (*A. pleuropneumoniae*) (*n* = 89) **(A)** and *Pasteurella multocida* (*P. multocida*) (*n* = 363) **(B)** by ECOFFinder. Insert graphs represent observed MIC distribution with MIC_90_.

The MIC of tylosin against 89 and 363 isolates of *A. pleuropneumoniae* and *P. multocida* strains spread widely, ranging from 1 to 256 μg/mL and from 0.5 to 128 μg/mL, respectively (Figures 1A, B), with a monomodal distribution displaying Gaussian distribution. Tylosin was active against both species isolates, with MIC_50_ and MIC_90_ of 16 μg/mL for *A. pleuropneumoniae* and MIC_50_ of 16 μg/mL and MIC_90_ of 32 μg/mL for *P. multocida*. Strains with MIC values similar to the MIC_90_ of tylosin against *A. pleuropneumoniae* (BA2000013) and *P. multocida* (BA1700127) were selected for further PD study. Both QC results were within the QC ranges specified by CLSI documents M100-S20. The MBC of tylosin against *A. pleuropneumoniae* and *P. multocida* was 32 μg/mL.

According to the EUCAST ECOFFinder analysis, the ECOFF value (≤64 µg/mL), 97.75% (87 strains) of the *A. pleuropnumoniae* isolates were wild-type, whereas with the same ECOFF value (≤64 µg/mL), 99.72% (363 strains) of the *P. multicoda* isolates were considered wild-type to tylosin using the indicated mode (Table 1).

**TABLE 1:** *A. pleuropneumoniae* and *P. multocida modes of MIC wild-type distribution and* ECOFF from pig isolate and mixed origins according to the EUCAST.

Isolates	Pig isolates from this study		Mixed origin EUCAST	
	Mode	ECOFF	Mode	ECOFF
*A. pleuropneumoniae*	16	64	32	64
*P. multocida*	16	64	32	ID^a^

^a^ Insufficient data.

### 3.2 PAE of tylosin

**FIGURE 2:**
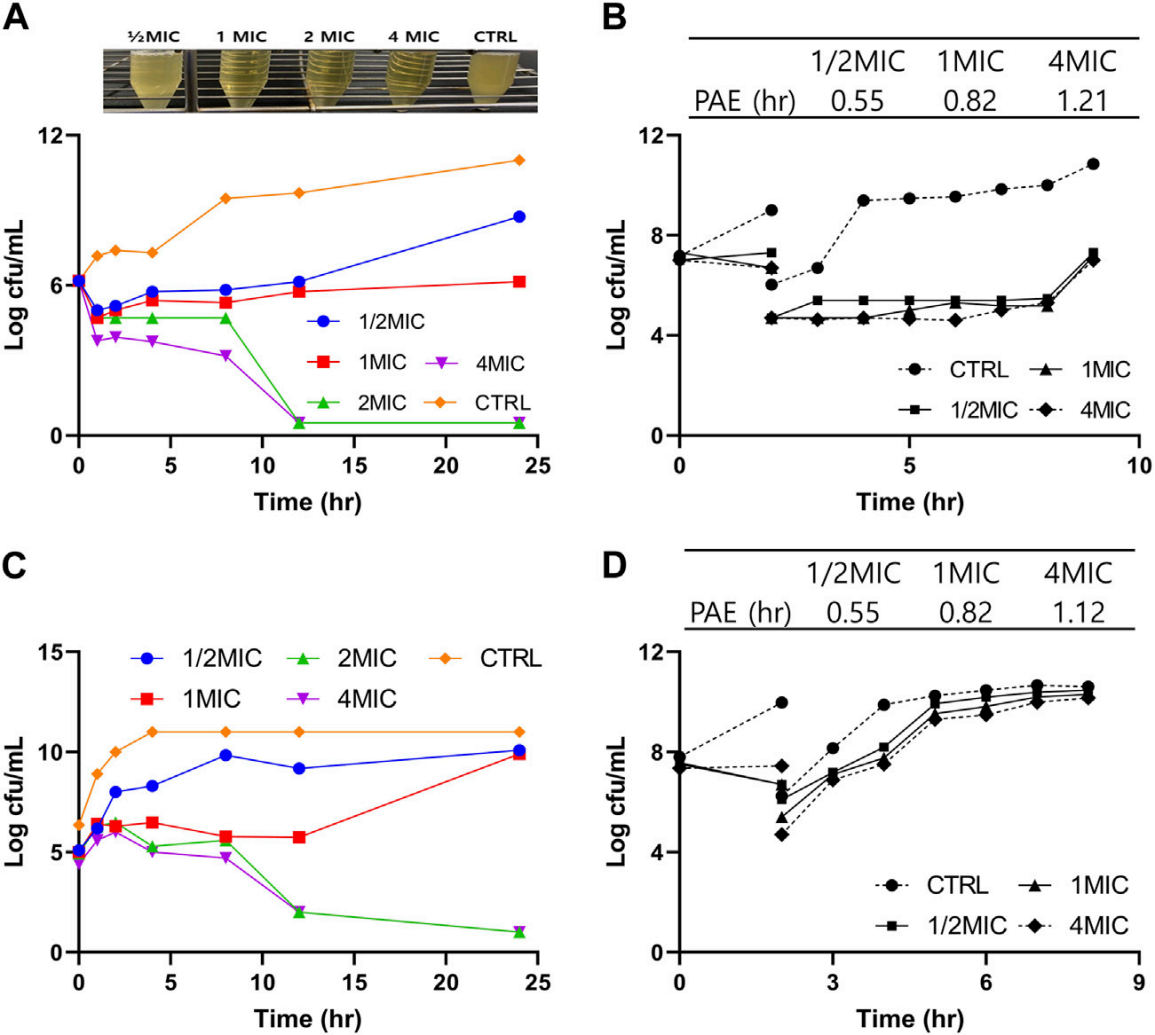
Pharmacodynamic (PD) study of tylosin against *A. pleuropneumoniae* and *P. multocida*. **(A)** *In vitro* time kill curves of tylosin against *A. pleuropneumoniae* at 0-, 1-, 2-, 4-, 8-, 12-, and 24-h time points and turbidity observations of tubes containing 1/2×, 1×, 2×, and 4× MIC of tylosin and control (without drug) following 24-h incubation, **(B)** post-antibiotic effect (PAE) following exposure to tylosin against *A. pleuropneumoniae*. **(C)** *In vitro* time kill curves of tylosin against *P. Multocida* at 0-, 1-, 2-, 4-, 8-, 12-, and 24-h time points. **(D)** PAE following exposure to tylosin against *P. multocida*.

The PAE against *A. pleuropneumoniae* following exposure to 0.5×, 1×, and 4× MIC concentrations lasted for 0.55, 0.82, and 1.21 h, respectively, following 2-h incubation (Figure 2B). Similarly, the PAE value against *P. multocida* lasted for 0.55, 0.82, and 1.12 h for 0.5×, 1×, and 4× MIC, respectively (Figure 2D). The results indicated that PAE has a positive relationship with exposure time.

### 3.3 *In vitro* and *ex vivo* time kill curves

**FIGURE 3:**
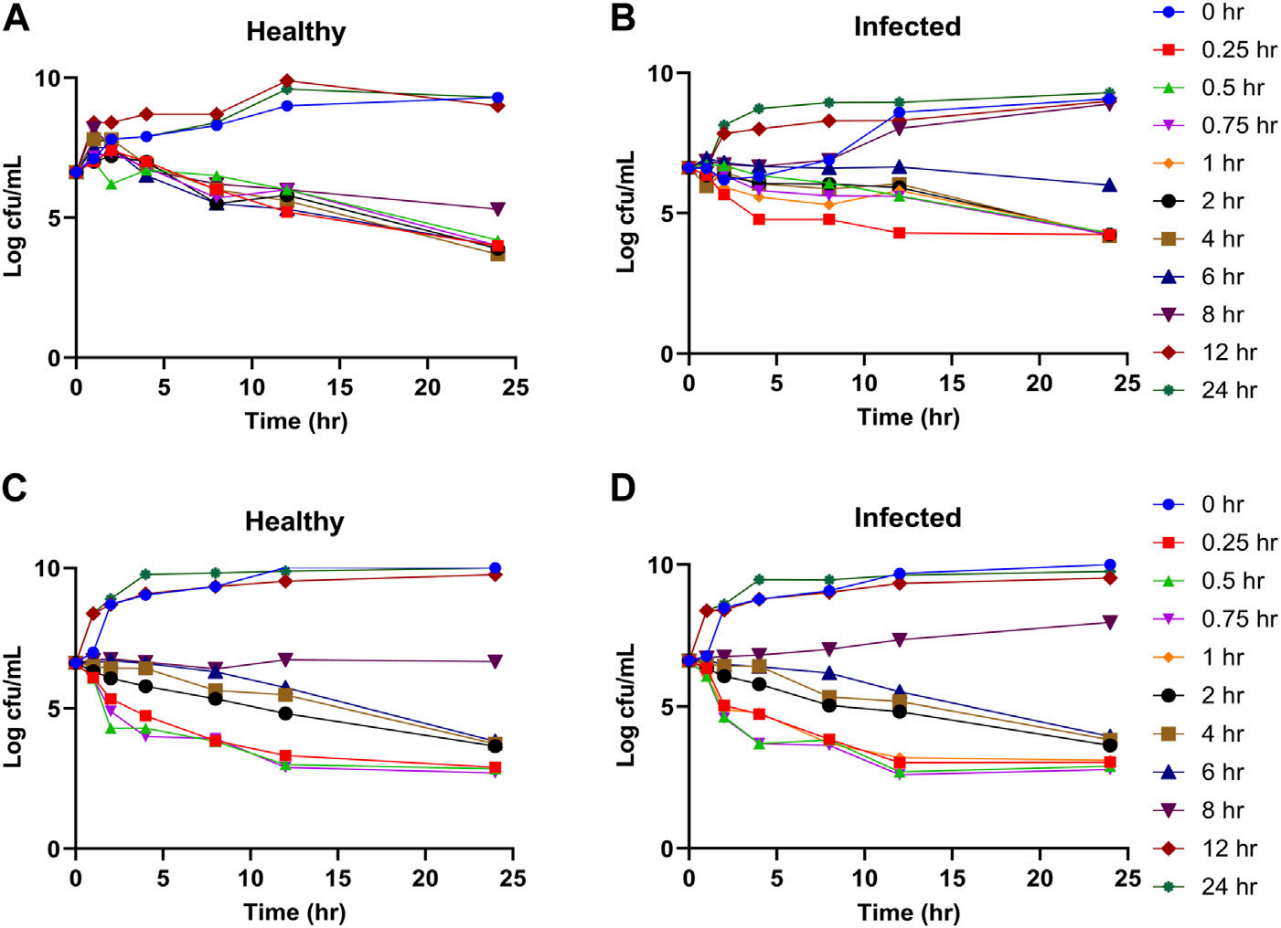
*Ex vivo* time kill curves of tylosin plasma against *A. pleuropneumoniae* (**(A)**, healthy; **(B)**, infected) and *P. multocida* (**(C)**, healthy; **(D)**, infected) at 0-, 1-, 2-, 4-, 8-, 12-, and 24-h time points.

The time kill curves of tylosin against *A. pleuropneumoniae* and *P. multicoda in vitro* and *ex vivo* are illustrated in Figures 2A, C, 3. According to the profiles of curves *in vitro*, increasing drug concentrations induced more rapid and radical bactericidal effects. The bacteria growth recovery observed following exposure to 1× MIC or less of tylosin fades away with markedly decreasing bacterial CFU values (<30 CFU) (Figures 2A, C) following exposure to a greater concentration than 1× MIC of tylosin for 24 h. Bacterial CFU values were also markedly decreased (<30 CFU) in the serum from pigs of the PK experiment from the infected and control groups for samples collected between 0.025 and 8 h (Figure 3). The time kill curves *in vitro* and *ex vivo* were analogical. These findings suggest that tylosin has a concentration-dependent action against *A. pleuropneumoniae* and *P. multicoda* both *in vitro* and *ex vivo*.

### 3.4 PK analysis of tylosin in healthy and infected pigs

Following 12 h of infection, infected pigs showed noticeable clinical symptoms, including depression, coughing, and a slight difficulty in breathing, compared with healthy pigs, thereby confirming a successfully established a co-infected pig model. Furthermore, body temperature in healthy and infected group was 37.2°C ± 1.5°C, 40.1°C ± 1.1°C (*p* < 0.001), respectively. Furthermore, *apxIVA* and *kmt1* genes for *A. pleuropneumoniae* and *P. multocida*, respectively, were confirmed positive using PCR target gene amplification with the size of 377 bp (Supplementary Figure S1A) and 460 bp (Supplementary Figure S1B) in infected pigs.

**FIGURE 4:**
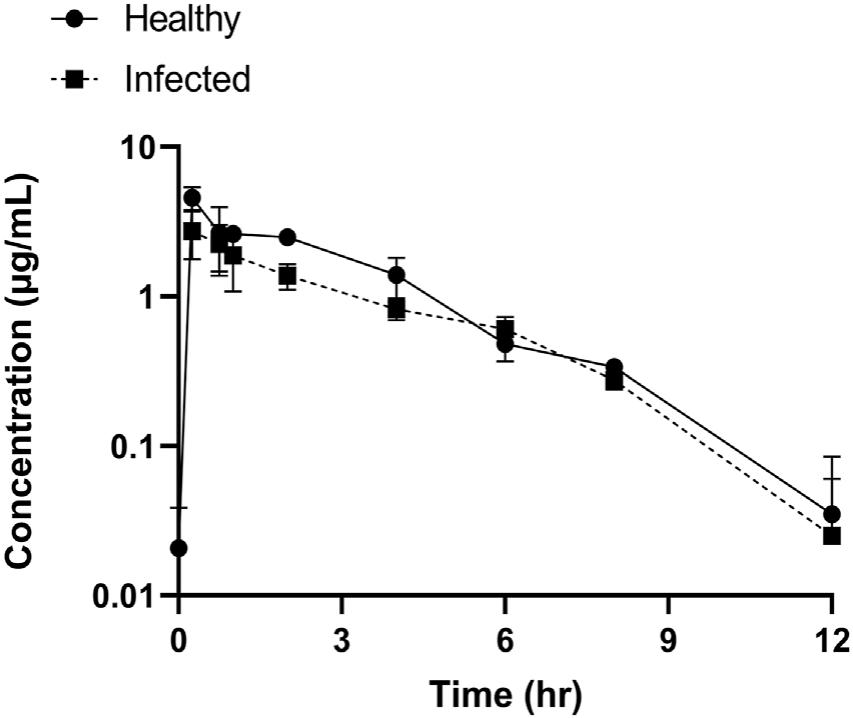
Plots of tylosin concentration–time in plasma following IM administration (20 mg/kg).

Tylosin plasma concentrations as a function of time profiles for the healthy and infected groups are presented in Figure 4. The outcomes achieved using blank samples were contrasted with tylosin-injected samples and no interfering peaks were detected.

The PK parameters of tylosin are presented in Table 2. The mean C_max_ of healthy and infected pigs were 5.79 and 3.59 μg/mL, respectively, with a T_max_ of 0.25 h in both groups. The AUC of healthy pigs (13.33 h μg/mL) was higher than that of infected pigs (10.46 h μg/mL) (*p* < 0.05). Differences in half-life were observed between healthy (1.99 h) and infected (1.83 h) pigs with a significant difference (*p* < 0.05).

**TABLE 2:** PK parameters of tylosin following IM administrations in healthy and infected pigs.

Parameters	Healthy	Infected
T_1/2_ (h)	1.99 ± 0.14	1.83 ± 0.51
T_max_ (h)	0.25 ± 0.00	0.25 ± 0.00
C_max_ (μg/mL)	5.79 ± 0.76	3.59 ± 0.66
AUC (h*μg/mL)	13.33 ± 0.78	10.46 ± 1.61
Vz/F (mL/kg)	4,045.83 ± 305.73	5,019.45 ± 2,147.48
Cl/F (mL/h/kg)	1,416.19 ± 100.04	1,905.66 ± 229.96
MRT (h)	2.47 ± 0.23	2.90 ± 0.07

T_1/2_ is half-life; T_max_ is the time to reach the maximum plasma concentration; C_max_ is maximum concentration in plasma; AUC_last_ is the area under the plasma concentration–time curve within 24 h; Vz/F is the apparent volume of distribution; Cl/F is clearance; MRT, is mean residence time; PK, pharmacokinetic; IM, intramuscular.

### 3.5 Sigmoid E_max_ model

As shown in Table 3, several essential PK/PD parameters are taken into account, including T > MIC, AUC/MIC, and C_max_/MIC (Nielsen et al., 2011). The bacterial killing effect of tylosin was shown through extended PAE as well as time killing curves, indicating its ability to effectively eliminate bacteria. This suggested that the ratio of AUC/MIC could be a beneficial PK-PD index to treat *A. pleuropneumoniae* and *P. multocida* infections.

**TABLE 3:** Parameters of PK/PD integration for tylosin against *A. pleuropneumoniae* and *P. multocida* following IM administration in pigs.

Parameter	*A. pleuropneumoniae*		*P. multocida*	
	Healthy	Infected	Healthy	Infected
AUC/MIC (h)	1.29 ± 0.39	1.01 ± 0.26	1.29 ± 0.39	1.01 ± 0.26
C_max_/MIC	0.30 ± 0.04	0.28 ± 0.04	0.30 ± 0.04	0.28 ± 0.04
T > MIC	13.08 ± 6.15	10.50 ± 6.51	6.54 ± 3.07	5.25 ± 3.26
E_max_	7.19 ± 0.72	7.34 ± 0.78	6.98 ± 0.34	7.04 ± 0.26
EC_50_	1.21 ± 0.15	1.33 ± 0.21	1.17 ± 0.05	1.06 ± 0.06
E_0_	2.84 ± 0.49	2.76 ± 0.39	3.49 ± 0.26	3.41 ± 0.18
γ	2.76 ± 0.90	1.86 ± 0.46	4.78 ± 1.12	2.90 ± 0.36
E_max_–E_o_	4.35 ± 0.23	4.58 ± 0.23	3.49 ± 0.07	3.63 ± 0.07
AUC_24 h_/MIC for bacteriostatic activity	0.98	1.03	1.1	1.12
AUC_24 h_/MIC for bactericidal activity	1.97	2.54	1.99	2.36

E_max_ is the maximum difference in bacterial counts; EC_50_ is the value to produce 50% of the maximal antibacterial effect; E_0_ is the maximal antibacterial effect; γ is the Hill coefficient.

**FIGURE 5:**
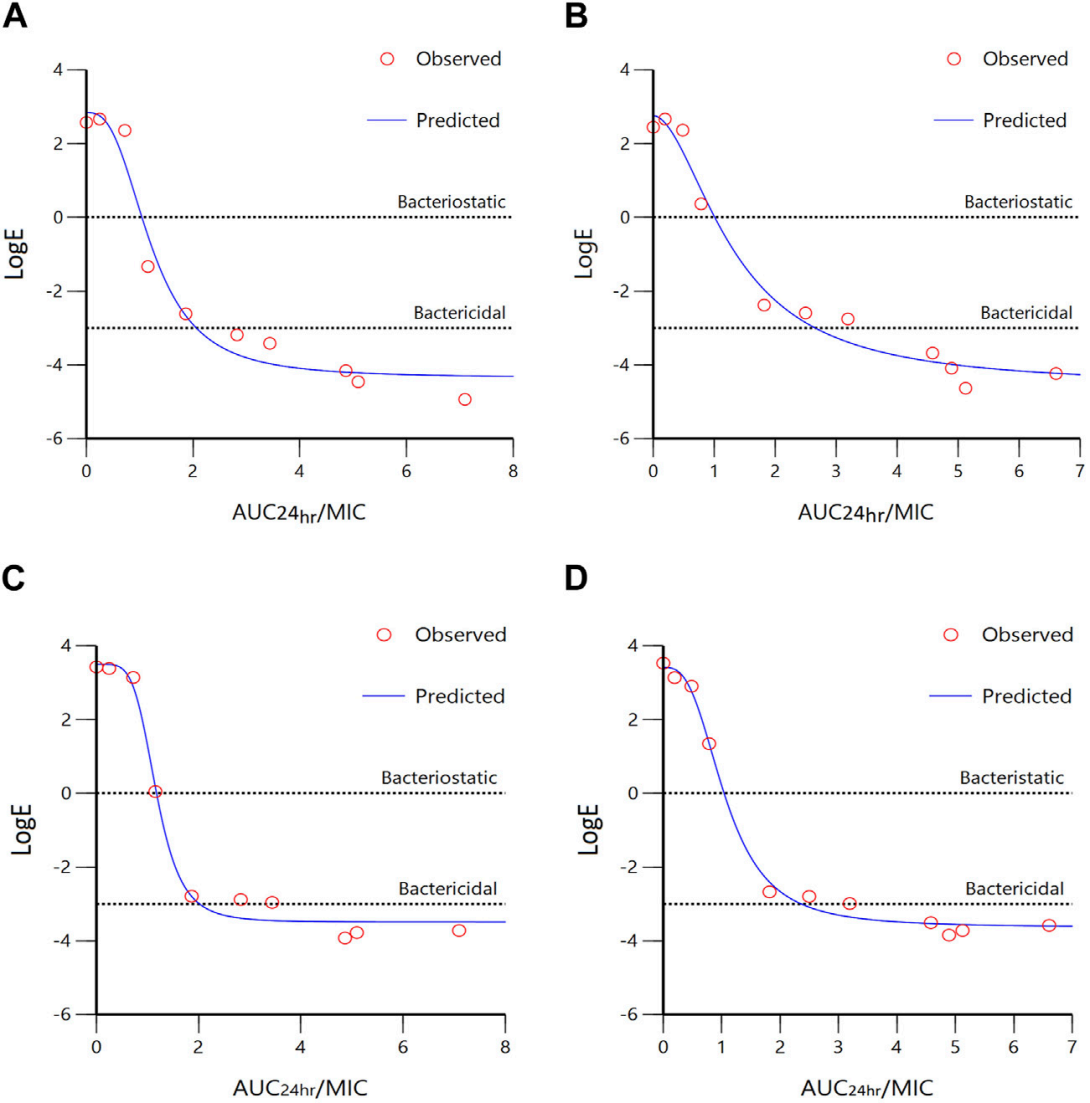
Sigmoidal Emax model of *ex vivo* AUC_24 h_/MIC ratios *versus* LogE (CFU/mL) of tylosin against *A. pleuropneumoniae* (**(A)**, healthy; **(B)**, infected) and *P. multocida* (**(C)**, healthy; **(D)**, infected) within 24 h. Dotted lines represent bacteriostatic (E = 0) and bactericidal (E = −3) activities.

Data obtained from simulating the Emax inhibitory sigmoid model for *A. pleuropneumoniae* are summarized in Figure 5; Table 3. The AUC_24 h_/MIC values for bacteriostatic and bactericidal activities in the plasma of healthy pigs were 0.98 and 1.97 h, respectively **(** Figure 5A). For infected pigs, 1.03 and 2.54 h were observed to produce bacteriostatic and bactericidal activities, respectively (Figure 5B). The E_max_ value of healthy pigs (7.19 ± 0.27) was slightly less than that of infected pigs (7.34 ± 0.78); however, this difference was not statistically significant.

The results of sigmoidal Emax models for *P. multocida* are summarized in Figure 5; Table 3. The AUC_24 h_/MIC values for bacteriostatic and bactericidal activities in the plasma of healthy pigs were 1.1 and 1.99 h, respectively (Figure 5C), whereas those for infected pigs were 1.12 and 2.36 h, respectively (Figure 5D). The E_max_ values were 6.98 ± 0.34 and 7.04 ± 0.26 for healthy and infected pigs, respectively, with no significant difference.

### 3.6 Dose estimation and CO_PD_ determination with MCS

**FIGURE 6:**
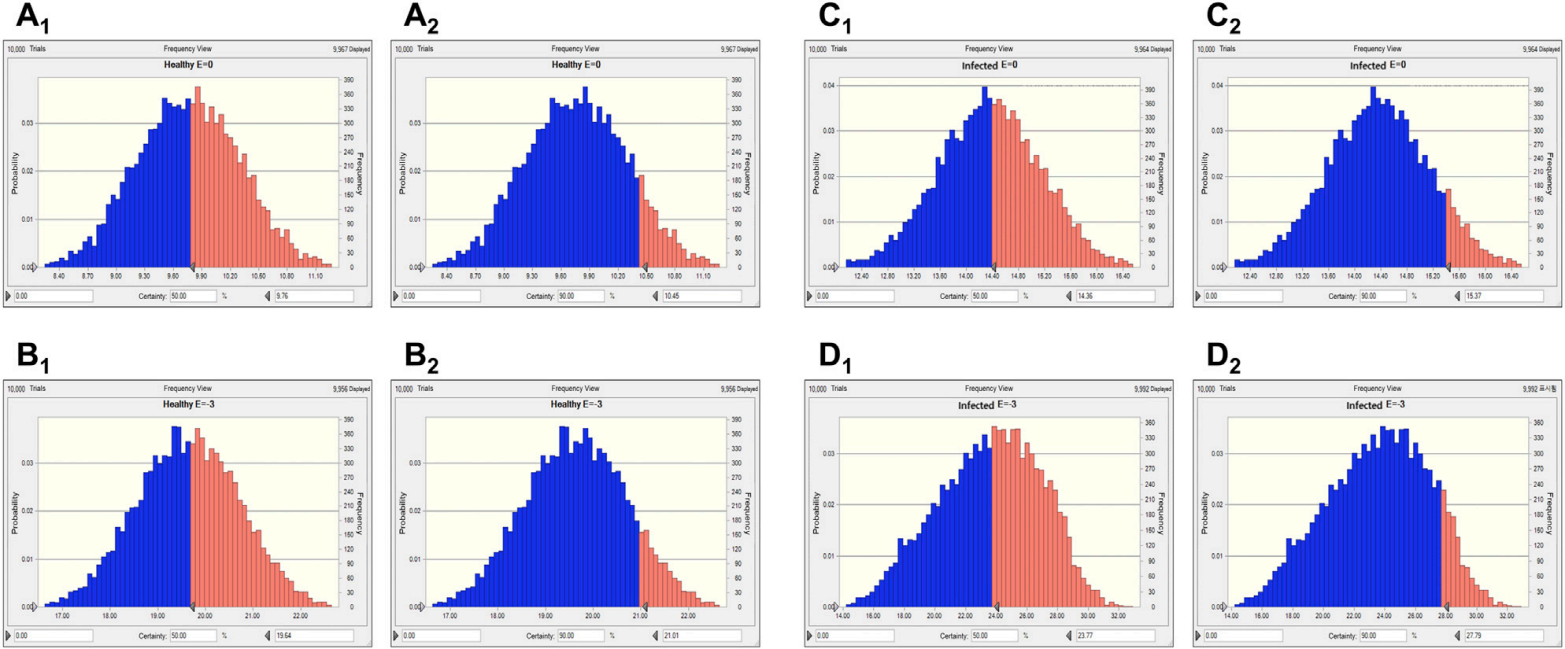
Predicted doses of tylosin for treating *A. pleuropneumoniae* at 50% and 90% target ratios in healthy and infected pigs. **(A**_**1**_ **)** The predicted population dose for the bacteriostatic activity at 50% target in healthy pigs; **(A**_**2**_ **)** the predicted population dose for the bacteriostatic activity at 90% target in healthy pigs; **(B**_**1**_ **)** the predicted population dose for the bactericidal activity at 50% target in healthy pigs; **(B**_**2**_ **)** the predicted population dose for the bactericidal activity at 90% target in healthy pigs; **(C**_**1**_ **)** the predicted population dose for the bacteriostatic activity at 50% target in infected pigs; **(C**_**2**_ **)** the predicted population dose for the bacteriostatic activity at 90% target in infected pigs; **(D**_**1**_ **)** the predicted population dose for the bactericidal activity at 50% target in infected pigs; **(D**_**2**_ **)** the predicted population dose for the bactericidal activity at 90% target in infected pigs.

**FIGURE 7:**
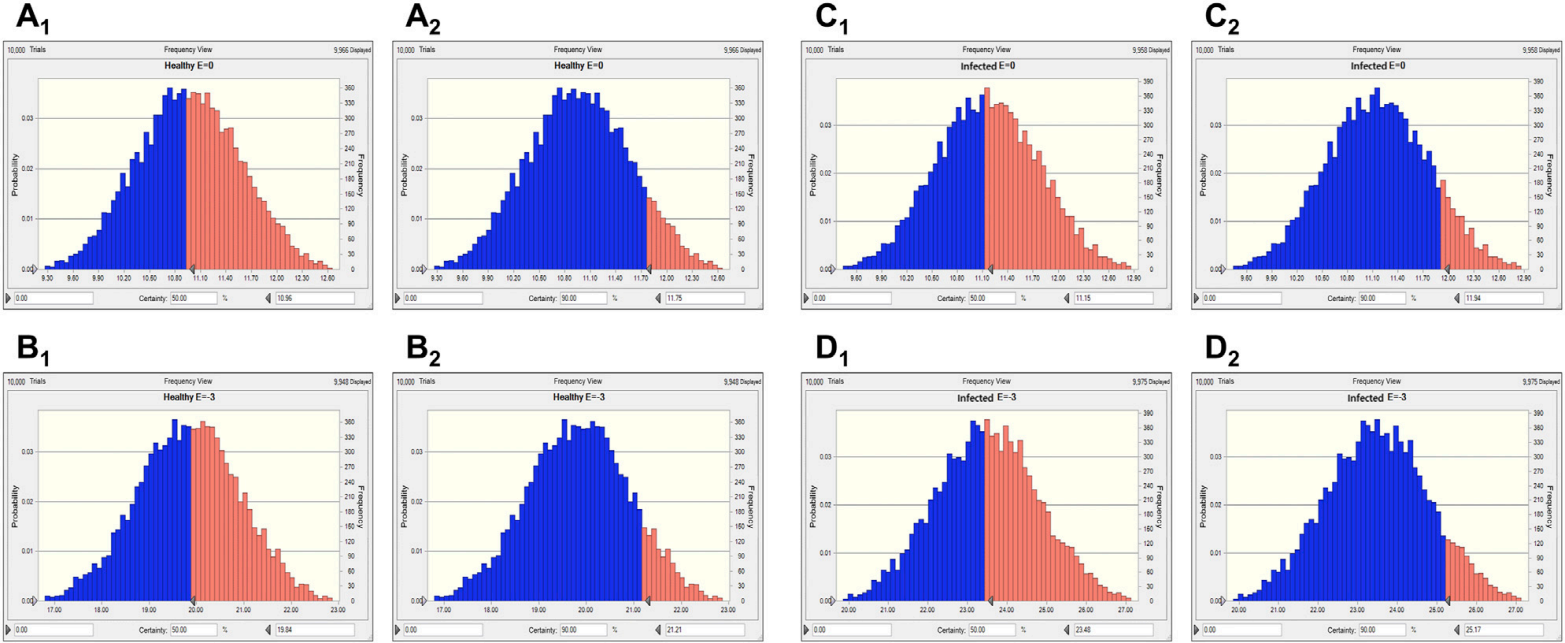
Predicted doses of tylosin for treating *P. multocida* at 50% and 90% target ratios in healthy and infected pigs. **(A**_**1**_ **)** The predicted population dose for the bacteriostatic activity at 50% target in healthy pigs; **(A**_**2**_ **)** the predicted population dose for the bacteriostatic activity at 90% target in healthy pigs,; **(B**_**1**_ **)** the predicted population dose for the bactericidal activity at 50% target in healthy pigs; **(B**_**2**_ **)** the predicted population dose for the bactericidal activity at 90% target in healthy pigs; **(C**_**1**_ **)** the predicted population dose for the bacteriostatic activity at 50% target in infected pigs; **(C**_**2**_ **)** the predicted population dose for the bacteriostatic activity at 90% target in infected pigs; **(D**_**1**_ **)** the predicted population dose for the bactericidal activity at 50% target in infected pigs; **(D**_**2**_ **)** the predicted population dose for the bactericidal activity at 90% target in infected pigs.

Two levels of antibacterial efficacy were determined by computing the AUC_24 h_/MIC value using PK/PD integration and *ex vivo* distribution, through MCS in the Oracle Crystal Ball software. The calculated doses for achieving the bactericidal activity of tylosin against *A. pleuropneumoniae* over 24 h were 21.01 and 27.79 mg/kg in healthy and infected pigs, respectively, for a 90% target, according to dose equations (Figure 6; Table 4). The predicted dosages for achieving the bactericidal activity of tylosin against *P. multocida* were 21.21 and 25.17 mg/kg in healthy and infected pigs, respectively, for a 90% target (Figure 7; Table 4). These results suggest that the optimal dosage of tylosin for *A. pleuropneumoniae* and *P. multocida* co-infections could be 25.17–27.79 mg/kg to achieve a bactericidal effect.

**TABLE 4:** Predicted daily doses of tylosin for treating *A. pleuropneumoniae* and *P. multocida*.

Predicted dose (mg/kg)			Target ratios	
			50%	90%
*A. pleuropneumoniae*	Bacteriostatic (E = 0)	Healthy	9.76	10.45
		Infected	14.36	15.37
	Bactericidal (E = −3)	Healthy	19.64	21.01
		Infected	23.77	27.79
*P. multocida*	Bacteriostatic (E = 0)	Healthy	10.96	11.75
		Infected	11.15	11.94
	Bactericidal (E = −3)	Healthy	19.84	21.21
		Infected	23.48	25.17

**FIGURE 8:**
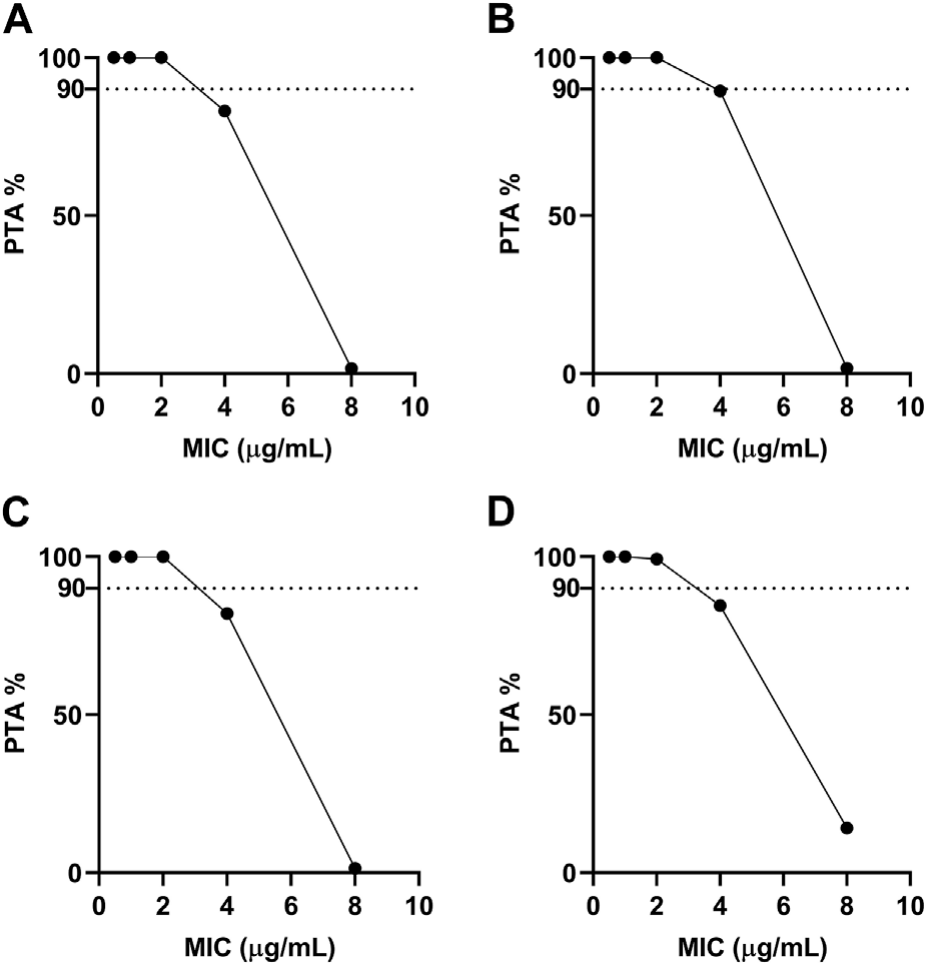
Probability of target attainment for treating with single-dose tylosin at 20 mg/kg for *A. pleuropneumoniae* (**(A)**, healthy; **(B)**, infected) and *P. multocida* (**(C)**, healthy; **(D)**, infected). Dotted lines represent 90% target.

Furthermore, MCS was conducted for 10,000 iterations using the Oracle Crystal Ball software for determining the CO_PD_ for tylosin target achievement calculations from *ex vivo* PD and PK data (Figure 8). Regarding the CO_PD_ for tylosin against *A. pleuropneumoniae,* the PTA was 83.13% at 4 μg/mL; however, a PTA of >90% was achieved when the MIC was <2 μg/mL in healthy pigs. The PTA for infected pigs was 100% at a MIC value of 2 μg/mL (Figure 8B). A similar pattern was observed for the CO_PD_ for tylosin against *P. multocida*, achieving >90% PTA at a MIC value of 2 μg/mL when the PTA was 100% and 99.25% in healthy and infected pigs, respectively. Therefore, the CO_PD_ for tylosin against *A. pleuropneumoniae* and *P. multocida* in both healthy and infected pigs was 2 μg/mL.

## 4 Discussion

Antibiotics are widely used in the pig industry for different purposes, including therapeutic, metaphylactic, and prophylactic (Lekagul, Tangcharoensathien, and Yeung, 2019). However, this practice has led to an increased risk of bacteria becoming resistant to these drugs (Holmer et al., 2019). A previous study has shown that *A. pleuropneumoniae*, which causes respiratory diseases in pigs, has developed resistance to several antibiotics, including tylosin (Aarestrup et al., 1998; Vanni et al., 2012). In this study, to ensure animal health and prevent the emergence of antibiotic resistance, determining the most effective treatment and establishing an appropriate dosage regimen are significant. A rational approach to dosing antibiotics, based on PK–PD modeling, can help maximize the benefits of treatment while minimizing the risk of developing resistance (Zhang et al., 2022).

Although macrolides are traditionally classified as bacteriostatic drugs, it is crucial to recognize that bacteriostatic antibiotics do have the capacity to kill bacteria; they simply require higher concentrations than bactericidal agents to achieve specific levels of bacterial reduction (Wald-Dickler et al., 2018). The effects of antibiotics can vary depending on the pathogens they target. For example, azithromycin has been shown to exhibit bactericidal activity against *Streptococcus pyogenes* (Piscitelli et al., 1992), whereas chloramphenicol demonstrates bactericidal activity against *Streptococcus pneumoniae* but acts as a bacteriostatic agent against *Staphylococcus aureus* (Turk, 1977; Rahal and Simberkoff, 1979). Previous study indicated that tylosin effectively eliminated *Streptococcus suis* in a both time and concentration manner, suggesting that the appropriate PK-PD index for tylosin is the AUC/MIC ratio (Huang et al., 2018). Therefore, macrolide do not fit into a single distinct category (T > MIC). In this study, the *ex vivo* time killing curves and prolonged of tylosin demonstrated its bactericidal effect, suggesting AUC/MIC could be a favorable PK-PD index for tylosin against both *A. pleuropneumoniae* and *P. multocida* infections.

*A. pleuropneumoniae* and *P. multocida* are one of the major contributors to porcine respiratory diseases worldwide. The symptoms of infection can include arthritis, breathing difficulties, and lethargy in young pigs (Jensen et al., 1999; Stringer et al., 2022). The incidence rates of *A. pleuropneumoniae* infection range from 25% to 48%, with a significant mortality rate, thereby causing significant economic losses in the industrial pig breeding industry (Vangroenweghe and Thas, 2021). Tylosin is frequently used for controlling *A. pleuropneumoniae* and *P. multocida*; however, there is limited information available on its PK parameters in livestock and poultry. From these perspectives, in the current study, we have established a PD profile of tylosin against 89 and 363 pig isolates of *A. pleuropneumoniae* and *P. multocida* strains, respectively, using *in vitro* and *ex vivo* assays and the PK profile of tylosin in healthy and infected pigs. Using these PK and PD profiles in an integrated PK–PD modeling, we developed an optimized dosage that can maximize tylosin treatment benefits in pigs (Toutain and Lees, 2004).

In the PD profile, the wild-type distribution of MICs of tylosin against *A. pleuropneumoniae* and *P. multocida* strains in the current study ranges from 0.5 to 32 μg/mL, with a similar scope to a previous study (Kim et al., 2001). The MIC_90_ values of 16 and 32 μg/mL of tylosin observed in the current study for *A. pleuropneumoniae* and *P. multocida*, respectively, were higher than those previously reported MIC_90_ values of 4 and 1 μg/mL of another macrolide gamithromycin against European isolates of *A. pleuropneumoniae* and *P. multocida*, respectively (EMA, 2015). Considering the bactericidal effects of tylosin, both species show MBC values that were close to their MIC. Tylosin shows a similar activity against both species with similar MBC and time kill curves with a slight indication of *A. pleuropneumoniae* likely to be the limiting pathogen in eradicating the bacteria in co-infections *in vivo* having a slightly higher MIC range.

We studied the PK of tylosin in both healthy and infected pigs with *A. pleuropneumoniae* and *P. multocida*. In particular, closely monitoring clinical signs, including body temperature, in both healthy and infected pigs is of utmost importance for the infected animals. These parameters provide valuable insights into the physiological changes during health and disease, especially regarding drug administration (Robbins et al., 2014). Body temperature is a critical indicator of an animal’s health status and helps assess the severity of infection and immune response (Teixeira et al., 2020). Additionally, measuring lung pH in both healthy and infected pigs is essential for understanding the pulmonary environment and its impact on drug behavior and treatment effectiveness (Bikou et al., 2018). Infections or respiratory diseases can alter lung physiology, resulting in changes in lung pH levels, which may influence drug uptake and efficacy, particularly for drugs targeting pulmonary infections (Torres et al., 2017).

PK profiles provide significant information about what the body does to the drugs (Rodríguez-Gascón et al., 2021). This information is essential for optimizing the efficacy and safety of a drug and designing effective dosing regimens that are needed to achieve a therapeutic effect (Koiwai et al., 2021). In this study, a single IM dose of 20 mg/kg of tylosin was administered, and PK parameters in the plasma, including C_max_, T_max_, AUC, T_1/2_, and MRT, were observed to be slightly different between healthy and infected pigs. In healthy and infected pigs, C_max_ of the drug in the bloodstream was found to be 5.79 μg/mL and 3.59 μg/mL, respectively. T_max_ was the same in both groups, occurring at 0.25 h after drug administration. However, AUC in healthy pigs (13.33 h·μg/mL) was higher compared to infected pigs (10.46 h·μg/mL), with a statistically significant difference (*p* < 0.05), indicating that healthy pigs had a greater overall exposure to the drug. Furthermore, there were differences in the drug’s half-life between healthy (1.99 h) and infected (1.83 h) pigs, with a significant disparity (*p* < 0.05). Moreover, higher clearance values in infected (1,905.66 mL/h/kg) was observed compared to healthy (1,416.19 mL/h/kg). This suggests that the drug persisted for a slightly longer duration in healthy pigs compared to infected pigs. The higher apparent volume of distribution in infected pigs (4,045.83 ± 305.73 mL/kg) compared with healthy pigs (5,019.45 ± 2,147.48 mL/kg) could be attributed to the physiological changes that occur during infection, which may lead to alterations in blood flow, tissue perfusion, and permeability (Smith et al., 2015). We attributed these observed differences in PK to the physiological condition of the animals, which was influenced by the presence of infection (Blot et al., 2014). The T_1/2_ value was 1.5-fold higher than that of a previous study (Huang et al., 2018) despite the dose used in this study being twice as high as in a previous study by (Huang et al., 2018) using 10 mg/kg. This suggests that tylosin has a slow clearance and sustained release, with a longer T_max_ and higher AUC. In other species, tylosin’s T_1/2_ value was reported as 2.88 h in ducks (Elazab et al., 2020), and 2.24 h in cattle (Saurit et al., 2002), 1.54 h in dogs (Kim et al., 2008) 0.54 h in cows (Avci and Elmas, 2014). This could be due to variances in animal physiology. Specifically tailored for time-dependent antibiotics, PK/PD integration centers on how drug concentration patterns throughout time influence the bactericidal activity against pathogens. Several essential PK/PD parameters are taken into account, including T > MIC, AUC/MIC, and C_max_/MIC (Nielsen et al., 2011). Previous research have reported the PK of other time dependent antibiotics which correlated with T > MIC (Ahmad et al., 2015; Sjölund et al., 2020). The PK of penicillin G was studied using a 3-compartment model, which included additional tissue compartments (Li et al., 2014). The central volume of distribution and central clearance were determined to be 3.05 L and 16.9 L/h, respectively. The peripheral clearance was found to be 0.52 L/h. The majority of drugs within this category are quickly removed from the body (Smith et al., 2018). Nevertheless, tylosin sets itself apart from other time-dependent antibacterial agents in certain aspects. This research demonstrated that the clinical effectiveness of tylosin with sustained release is influenced by AUC_24h_/MIC ratio, suggesting distinctions not just in its T_1/2_ but also in how it penetrates tissues and subsequently releases the antibiotic (Van Bambeke and Tulkens, 2001).

The integration of PK/PD principles plays a vital role in comprehending the connection between drug concentration and its pharmacological effects (Zhang et al., 2022). This strategic PK/PD combination enables us to fine-tune dosing schedules and attain the most favorable therapeutic results (Rodríguez-Gascón et al., 2021). To investigate the antibacterial effects of tylosin against *A. pleuropneumoniae*, a PK/PD integration model using the inhibitory sigmoid Emax model was employed. This model demonstrated a strong correlation (*R* ^2^ = 0.99) between the observed and predicted efficacy of tylosin against *A. pleuropneumoniae*. The findings indicated that AUC_24 h_/MIC has the potential to serve as the PK-PD index for this particular model. Infected pigs had individually higher AUC_24 h_/MIC values required for bacteriostatic and bactericidal effects against *A. pleuropneumoniae* and *P. multocida* than healthy pigs. This result indicates the significance of using clinical PK parameters (PK from infected pigs) in determining the optimal dosage regimen. Based on the AUC_24 h_/MIC values for bacteriostatic (Healthy: 0.98 h, Infected: 1.03 h) and bactericidal (Healthy: 1.97 h, Infected: 2.54 h) activities against *A. pleuropneumoniae* and, bacteriostatic (Healthy: 1.1 h, Infected: 1.12 h) and bactericidal (Healthy: 1.99 h, infected: 2.36 h) activities against *P. multocida*, desirable dosage was determined. Our study showed that the accurate doses of tylosin for a PTA of ≥90% are 10.45–11.75 and 11.94–15.37 mg/kg for healthy and infected pigs, respectively, which would be sufficient for a bacteriostatic effect against *A. pleuropneumoniae* and *P. multocida* co-infections while 21.01–21.21 and 25.17–27.79 mg/kg for healthy and infected pigs could have bactericidal effect against *A. pleuropneumoniae* and *P. multocida*. However, since PK and PD data were obtained from a small sample size, these predicted daily dosages must be validated in clinical practice.

Potential adverse effects linked to the administration of high doses of drugs to animals may involve gastrointestinal irritation, resulting in symptoms such as diarrhea, vomiting, or other gastrointestinal problems (Makins and Ballinger, 2003). While no previous research on adverse effects after intramuscular administration of tylosin to pigs has been reported, studies have demonstrated that tylosin exhibited low acute oral toxicity in rats, mice and dogs. The LD50 values in rats and mice are in excess of 5,000 mg/kg, and in dogs, it is greater than 800 mg/kg. Overt signs of toxicity observed in dogs included salivation, vomiting, and defecation (CVMP, 1997). Moreover, the frequency of administration and redosing intervals when dealing with wild type strains beyond the MIC distribution can be considered for future investigations. This is especially crucial as tylosin’s effectiveness can be influenced by various factors, including dosage, frequency of administration, and the specific bacterial strain being treated, as observed in previous research (Wu et al., 2019).

To determine the susceptibility and resistance of bacteria, a clinical breakpoint is frequently used (Humphries, Abbott, and Hindler, 2019). To establish this breakpoint, factors, including ECOFF, CO_PD_, and clinical cutoff values, should be considered (Espinel-Ingroff and Turnidge, 2016). The CO_PD_ value for tylosin against *A. pleuropneumoniae* and *P. multocida* was 2 μg/mL, which is lower than the ECOFF value of *A. pleuropneumoniae* and *P. multocida*. This suggests that the current dose of 20 mg/kg may be insufficient for treating wild-type populations. While most studies (Boothe et al., 2002; Avci and Elmas, 2014; Lee et al., 2021) have primarily focused on the drug concentrations in serum/plasma for PK/PD studies, it is crucial to consider the interstitial tissue as it is commonly invaded by most bacteria. Evaluating the antibiotic concentration in the interstitial fluid of the target tissue becomes essential for assessing the antibacterial effects (Matzneller et al., 2013). PD parameters based on plasma concentration of macrolide antibiotics may not be suitable for managing respiratory infections due to much higher concentrations in the respiratory tract compared to serum/plasma (Drusano, 2005). Generally, the plasma concentrations of macrolide antibiotics in animals, even after administering recommended doses, remain notably lower than their respective MIC value (Rose et al., 2013). Therefore, measuring interstitial fluid concentration of tylosin should be considered to support the valid MIC value for clinical application.

The study has several limitations. One limitation of the study is the absence of direct measurements of lung pH. While we considered various physiological parameters, including body temperature, to gain insights into the impact of infection on drug behavior and efficacy, the lack of lung pH measurements is a potential gap in understanding the pulmonary microenvironment’s influence on drug response. As mentioned earlier, infections or respiratory diseases can lead to changes in lung pH levels, which can significantly affect drug uptake and effectiveness, especially for drugs targeting pulmonary infections (Bikou et al., 2018). Including direct measurements of lung pH could have provided more comprehensive data and a clearer understanding of how the drug behaves in the lungs of infected animals, thus enhancing the accuracy of the dosage regimen for tylosin in clinical settings (Schanker and Less, 1977; Taburet et al., 1990).

In this study, the PAE against *A. pleuropneumoniae* was observed after exposure to 0.5×, 1×, and 4× MIC concentrations, lasting for 0.55, 0.82, and 1.21 h, respectively, following a 2-h incubation. Similarly, for *P. multocida*, the PAE durations were 0.55, 0.82, and 1.12 h at 0.5×, 1×, and 4× MIC, respectively. However, there is no data available regarding the PAE against co-inoculation of A. *pleuropneumoniae* and *P. multocida*. Bacteria engage in interactions within their own species, with different species, and sometimes across entirely different genera, families, or even domains (Weiland-Bräuer, 2021). The presence of multiple pathogens in co-infections could have a significant impact on either improving or worsening disease outcomes (Devi et al., 2021). Co-infection has the potential to either extend or shorten the PAE. It is crucial to understand how co-infections influence the PAE, as this knowledge is vital for managing complex bacterial infections and developing effective treatment strategies. Further research can explore the intricate interplay between these strains and its implications for disease severity and treatment outcomes.

The impact of the immune response and the size of the bacterial inoculum on treatment outcomes should be considered (Li et al., 2017). Mice were exposed to *Citrobacter rodentium* to investigate how tylosin influence host responses to physiological stress (Brown et al., 2016). Tylosin treatment led to a decrease in the expression of antimicrobial peptide (β-defensin 1) and helper T cell 17 cytokine (interleukin-17a) in the intestine. Conversely, it demonstrated a significant increase in the levels of interleukin-17a and regulatory T cell cytokine (interleukin-10). In addition to its direct antibacterial effects against mycoplasmosis, tylosin seems to have an additional advantage as it enhances cell-mediated immune responses in chickens (Baba et al., 1998). Taking into account that the immune response can influence the effectiveness of tylosin treatment, conducting additional research on the immune response to tylosin could provide valuable support for the findings related to dosage optimization. *In vitro* studies have extensively examined the impact of inoculum size on antibacterial activity (Athamna et al., 2004). However, there are limited reports discussing the influence of inoculum size on the *in vivo* efficacy of antimicrobial agents (Chuang et al., 1998). Previous research indicated that the *in vitro* antimicrobial activity and *in vivo* efficacy of fluoroquinolones were minimally affected by the inoculum size, unlike carbapenems. This suggests that the reduced bactericidal activity or *in vitro* PAE of carbapenems and fluoroquinolones might be linked to their diminished *in vivo* protective effect against infections caused by high bacterial inocula of *S. aureus* or *Pseudomonas aeruginosa*. These findings could provide valuable insights into assessing the efficacy of antimicrobial agents in other animal infections. In the current study, the optimal dosage of tylosin was obtained with an inoculum size of 2.0 × 10^9^. However, it is essential to tailor treatment strategies based on the specific bacterial burden to enhance the likelihood of successful therapeutic outcomes. Considering the bacterial inoculum size in treatment decisions can be crucial for optimizing antimicrobial efficacy and combating infections effectively in animals.

In conclusion, the present study has shown that the PK parameters of infected animals are representative of clinical conditions and can be useful in designing optimal drug dosage regimens. Therefore, the current study on the PK characteristics of tylosin in healthy and infected pigs with *A. pleuropneumoniae* and *P. multocida* is of practical significance. The CO_PD_ (μg/mL) value determined in our study holds greater importance and practical relevance in preventing the emergence of resistance compared to the ECOFF value. Furthermore, tylosin could be a valuable treatment option for effectively managing pigs co-infected with *A. pleuropneumoniae* and *P. multocida*. A carefully selected dosage regimen of 11.94–15.37 mg/kg can achieve the desired bacteriostatic activity, while a dosage of 25.17–27.79 mg/kg is determined to achieve bactericidal effect. Nevertheless, the influence of the immune response and the size of the bacterial inoculum on treatment outcomes must be taken into account (Li et al., 2017). To validate and optimize the usage of tylosin in veterinary settings, further research is necessary to evaluate the most effective dosage for treating infected pigs.

## Data Availability

The original contributions presented in the study are included in the article/Supplementary Material, further inquiries can be directed to the corresponding authors.
