# Supplementary figures and images for: Optimizing tylosin dosage for co-infection of Actinobacillus pleuropneumoniae and Pasteurella multocida in pigs using pharmacokinetic/pharmacodynamic modeling
Source: Front Pharmacol. 2023 Sep 22;14:1258403. doi: 10.3389/fphar.2023.1258403 (PMC10556534; doi:10.3389/fphar.2023.1258403)

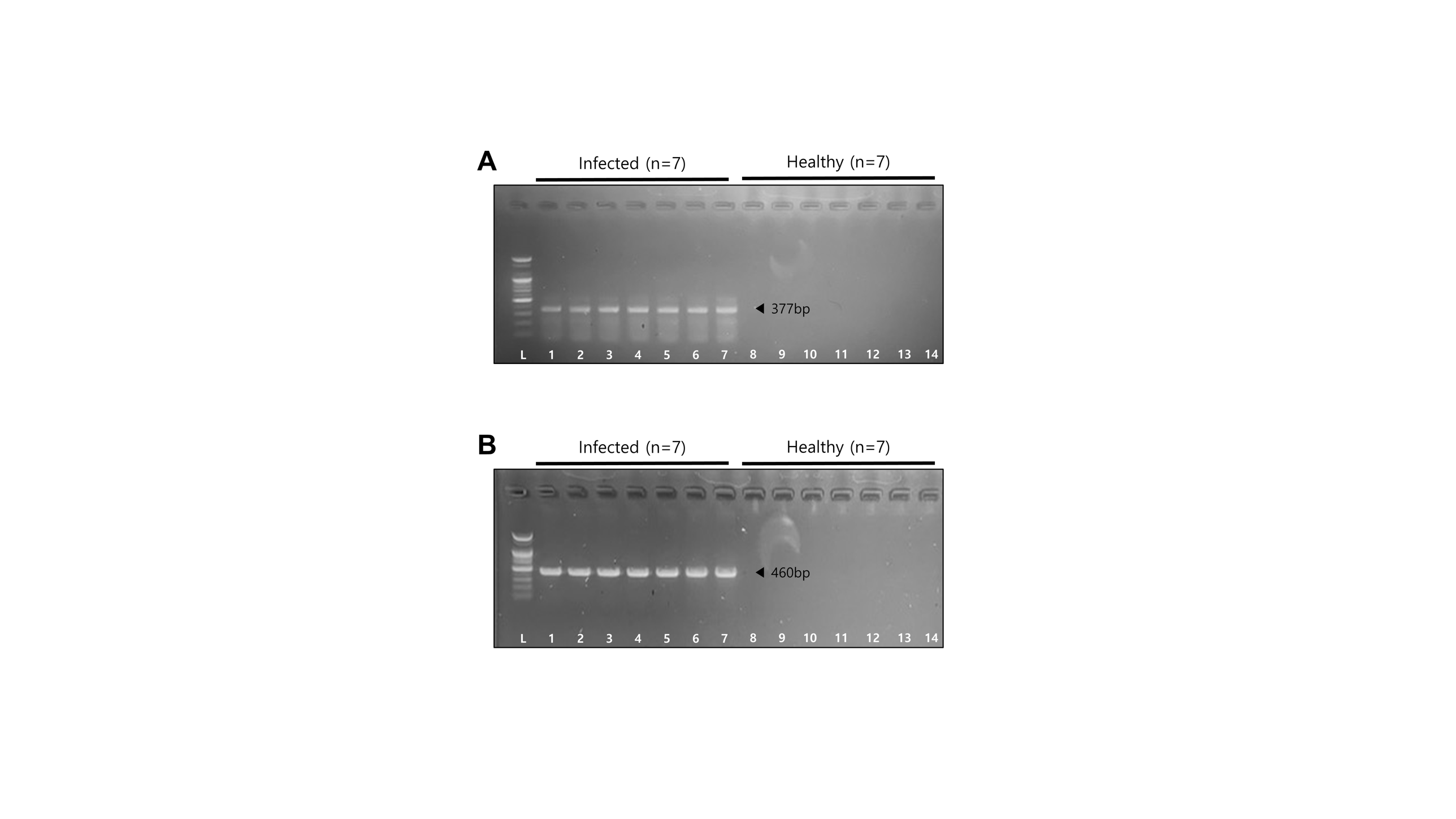

Supplement: Supplementary file 1 [file Image1.JPEG]
